# Untying the Influence of Green Brand Authenticity on Electronic Word-of-Mouth Intention: A Moderation–Mediation Model

**DOI:** 10.3389/fpsyg.2021.724452

**Published:** 2021-09-03

**Authors:** Yuhuan Xu, Jianguo Du, Fakhar Shahzad, Xingwei Li

**Affiliations:** ^1^School of Management, Jiangsu University, Zhenjiang, China; ^2^Department of Business Administration, ILMA University, Karachi, Pakistan; ^3^College of Architecture and Urban-Rural Planning, Sichuan Agricultural University, Chengdu, China

**Keywords:** green brand authenticity, electronic word-of-mouth, brand trust, self-concept consistency, green development

## Abstract

Green brands have made considerable strides in recent years; however, their validity has been questioned due to green brand fraud. However, the influence of green brand authenticity on consumer online behavior is still lacking in the e-commerce boom era. This article presents a theoretical framework based on trust and self-determination theory to investigate the influence of green brand authenticity on electronic word-of-mouth (eWOM). The conclusions are drawn from an empirical examination of 292 valid responses. Green brand authenticity influences eWOM intent, which is mediated through brand trust. Self-concept consistency has a moderating effect on the relationship between green brand authenticity and brand trust. The findings paved the way for future green brand development, notably in terms of publicity and promotion. This article also describes its theoretical and management significance, limitations, and future research directions.

## Introduction

Environmental issues are growing in importance as global warming continues to worsen, the main hindrance to global progress. According to the World Bank data, economic losses across the globe caused by various environmental pollutions reached 2.8% of gross national income in 2018 (UNEP, 2011), leading to a growing call for a green economy as an ideal way for advancing the economy. Additionally, based on direct losses recorded by international businesses, we estimate that the entire loss of consumption, including spillovers across all regions and industries, is approximately $3.8 trillion, or 4.2 percent of global gross domestic product (Lenzen et al., 2020). Featured by low carbon, high resource efficiency, and inclusive development, the green economy is aimed to achieve green growth (Li et al., 2018, 2019a), which could be difficult to realize if without environment-friendly technologies and products (Li et al., 2020; Mealy and Teytelboym, 2020), notably green brand. The green brand has made headway in recent years, and it clearly has a competitive advantage over non-green brands, as it supports sustainable development in a healthier, safer, and more sustainable way (Cinelli and LeBoeuf, 2020). However, the development of a green brand is not smooth sailing, as evidenced by the lack of ability for consumers to distinguish which brand is “green.” The chances are that they might run into green brand fraud problems with an increasing number of brands claim themselves as “green brand.” As a result, they would eventually lose trust in the green brand and focus on brand authenticity, or their perception of the brand’s reliability, such as quality, as a basis for deciding whether to be more friendly with it (Guèvremont, 2018). Some scholars argued that brand authenticity could bring about positive brand characteristics of persistence and originality (Busser and Shulga, 2019; Durkan, 2020), but whether green brand authenticity could be improved to restore the lost brand trust remains to be studied.

According to the cognitive-behavior theory (Aslin and Rothschild, 1987), when consumers perceive a green brand’s authenticity, they will act on that brand. The vigorous development of e-commerce has accelerated consumers’ deviation from the traditional way of offline shopping. People will naturally turn to the Internet to express their feelings about the validity of green brands using electronic word-of-mouth (eWOM). The eWOM, filled with both the consumers’ positive and negative comments, is also commonly referred to as online review, online recommendation, or online opinion (Serra Cantallops and Salvi, 2014). The study of Litvin et al. (2008) pointed out that interpersonal influence and word-of-mouth (WOM) tends to be some of the most important sources of information when consumers make decisions. As a result of eWOM, most young people have purchased things that are widely popular on social media (Cinelli and LeBoeuf, 2020). In this context, studying the impact of the authenticity of green brands on eWOM intentions is conducive to the promotion of green brands and environmental protection efforts, thereby forming a healthy and green development model. The functional relationship between green brand authenticity and eWOM intention remains largely unclear.

Brand managers are not the only ones liable for constructing the brand tryst and its value; several other aspects assist in creating the green brand authenticity positively, such as brand trust and self-interest consistency. For brand managers, in the pervasive presence of eWOM, establishing an image of green brand authenticity for any brand is the biggest challenge. Brand managers are required to create a positive brand image in the current digital environment (Chaudhuri and Holbrook, 2001; De Vries and Carlson, 2014). Even if the brand is real, it does not mean that the brand is successful because, for some consumers, the brand can represent a real brand, while for other consumers, the brand can have some other connotation, which should be related to emotion (Lude and Prügl, 2018; Cinelli and LeBoeuf, 2020). Sometimes, the market’s diagnosis of authenticity is a crucial prerequisite for the product’s release since it gives consumers a base for comfort with the product’s genuine qualities, despite the company’s artificial efforts (Moulard et al., 2016). At present, the millennial has a greater degree of affection for the brand. When the current era retains the green authenticity of the brand, they launch new models or modify the product style according to the market demand. In fact, they did not diverge from the real brand (Ewing et al., 2012).

In this context, self-determination theory proposes: (a) People are born to internalize the adjustment of important boring activities; (b) internalization will produce different self-regulation methods; and (c) social environment affects the internalization process and supervision methods (Deci et al., 1994). That suggests that people’s ability to regulate their emotions in response to the same stimulus can differ from one another. The extent to which an individual’s self-perception is consistent with their perception of things (in this case, green brand) is generally referred to as self-concept consistency. Then, do consumers with different self-concept consistency have different levels of trust in the brand? Is there a pattern to its effects? Although the importance of brand authenticity has become a consensus, many scholars have limited their research on how consumers judge whether a product is genuine (Cinelli and LeBoeuf, 2020) or identifying a product as being environmentally friendly (Mealy and Teytelboym, 2020). The research on authenticity is still in its infancy (Busser and Shulga, 2019), and its empirical research is still lacking (Moulard et al., 2016). Research on brand authenticity has primarily focused on generic brand determinants, such as brand story, perceived value and trust, brand attachment, and brand loyalty (Malär et al., 2011; Dangelico and Vocalelli, 2017; Bakshi and Mishra, 2018; Cinelli and LeBoeuf, 2020; Huang and Guo, 2021), rather than on specific brand determinants, such as green brand authenticity and eWOM intentions.

Research on green brand authenticity and eWOM intention regarding brand trust and its characteristics, especially the strict requirement on environmental protection, is still under-explored (Eggers et al., 2013). We further need to determine whether buyers value the green brand authenticity and are apprehensive of amicable interactions with green brands. Given that there is little research on the influence mechanism of green brand authenticity on eWOM intention, this paper is intended to fill in this gap by identifying the relationship between green brand authenticity and eWOM. Based on the above, this paper answers the following questions: (1) What is the role of green brand authenticity in developing eWOM intention? (2) does brand trust play a mediating role between the authenticity of the green brand and eWOM intention? and (3) does self-concept consistency have a moderating effect on the mediating effect of brand trust? These questions have important theoretical and practical significance for enriching green brand authenticity and eWOM intention. This paper aims to reveal the influence mechanism of green brand authenticity on eWOM intention and find out the internal relationship. In this paper, a moderation–mediation model is constructed and tested by empirical methods.

This study’s structure comprises the following section describing the theoretical review of the relevant literature and the development of hypotheses. The research method is in Section “Research Methodology,” and the research results are discussed in Section “Results and Discussions.” Conclusions, impacts, limitations, and prospects are given in Section “Conclusion, Implications, Limitations, and Future Prospects” and last the references.

## Theoretical Review and Hypothesis Development

The idea of authenticity can be used as a boundary requirement for consumer evaluation and behavior (Akbar and Wymer, 2017). Perceived authenticity plays a key role in the decision-making process of consumers’ brand and repurchase intention (Lude and Prügl, 2018). Over the past decade, a growing quest for authenticity in utilization can be observed in response to market homogeneity. Although the term authenticity has been studied in depth in philosophy, psychology, sociology, management, and many other disciplines, the definition of the concept varies from field to field (Fritz et al., 2017). A considerable amount of research has focused on brand authenticity in marketing, which has long been seen as an integral part of a brand (Schallehn et al., 2014; Kim and Hall, 2015; Moulard et al., 2016; Huang and Guo, 2021). However, it is not enough to simply develop authentic brands and products; the item must also be viewed as authentic by the brand’s target consumers. The real and the unreal stem from consumers’ recognition and interpretation of things that needs to be considered (Grayson and Martinec, 2004; Napoli et al., 2016). In the context of green marketing, green products’ authenticity lies in the subjective perception of consumers (Li et al., 2019b). Therefore, this article underlines the constructive view of green brand authenticity and eWOM, consumers’ subjective evaluation of brand authenticity.

The authenticity of a brand is based on its originality, stability, and consistency with the core values of the brand (Fritz et al., 2017). A study by Grayson and Martinec (2004) classifies authenticity into indexical authenticity and iconic authenticity. The former refers to the original product, which is different from the counterfeit goods, while the latter refers to the similarity of the object’s physical representation. This is the brand authenticity understood from the objective attribute, but this paper takes the consumer as the research perspective. In order to facilitate consumers’ understanding, we believe that brand authenticity is a kind of subjective perception that combines the inherent attributes of a brand with consumer’s own experience and knowledge (Morhart et al., 2013), that is, consumer’s assessment of the authenticity of the brand image, which serves as the basis for judging whether to conduct friendly interaction with the brand. However, experts have yet to explore the value of brand authenticity in the digital age, when consumers’ social media behavior is significantly emphasized (Arya et al., 2019). Similarly, fostering trust in green brands fosters positive involvement, which may have an effect on consumer intentions.

Authenticity has been studied in various ways in the past. As per the research by Ewing et al. (2012), the authenticity of a particular brand can be formed by certain types of authenticity clues. Firstly, the green brand boasts unique characteristics that other brands do not share, notably its green nature. Secondly, quality stands at the core of product competition. Brand authenticity will enhance consumer expectancy of product quality (Cinelli and LeBoeuf, 2020). It is frequently the case that a product’s perceived quality gives it an advantage over its competitors when it comes to consumer preference, since consumers naturally feel that brand authenticity implies product quality consumer (Han et al., 2019). Nowadays, the consumer-brand connection process has strengthened, which helps both parties. Social media platforms increase users’ curation, innovation, and collaboration options, hence enhancing customer trust (Eggers et al., 2013; Arya et al., 2019). Authenticity is related to consumers’ feelings and emotions in the postmodern era, rather than with their knowledge or truth (Phung et al., 2019). For example, it is difficult for consumers to establish whether organic food is chemical-free; thus, trusting the product’s authenticity is critical (Huang and Guo, 2021).

In addition, consumers’ perception of organizational transparency is a prerequisite for brand authenticity (Busser and Shulga, 2019). The perception of organizational transparency also influences the consumer’s judgment on whether the brand is operating honestly, which means that brand integrity is also a prerequisite for consumers to perceive brand authenticity. Therefore, green brand authenticity should include green attributes authenticity, quality commitment authenticity, and integrity authenticity. Recent studies focused on precursors and consequences of perceived authenticity in several research areas (Hede et al., 2014; Ye et al., 2018; Chen et al., 2020; Komarac et al., 2020). However, the concept of green brand authenticity remains sparse in the prior research, which needs to be examined on the relationship of green brand authenticity and eWOM intention. The authors of this study examined the effects of green brand authenticity on eWOM intention, as evaluated by green attributes, quality, and integrity.

Based on the trust theory and self-determination theory, this article proposed a theoretical model from consumers’ perspectives and provided a theoretical basis for the follow-up research. From the practical point of view, the exploration of the authenticity of green brands is conducive to consolidating the competitive position of green brands in the market, achieving market success and reputation, and becoming a trustworthy environmental brand (Huang and Guo, 2021). By filling the existing gap in this particular field, the results of this paper can also provide strategic insight to understand the research of green brand authenticity in engendering and facilitating eWOM intention, which is conducive to further developing green brands by virtue of eWOM on the electronic platforms, to alleviate the current situation of low market share of the green brand.

### Green Brand Authenticity and eWOM Intention

Brand authenticity has been frequently connected to brand trust (Eggers et al., 2013). In essence, authentic brands are devoted to delivering on their promises (Morhart et al., 2013; Fritz et al., 2017), and consumers trust brands that perform as promised (Napoli et al., 2016). Trust is declining in today’s consumer skepticism, and authenticity provides just the antidote for this (Fritz et al., 2017). The key elements of brand authenticity are “individuality,” “originality,” and “naturalness” (Schallehn et al., 2014; Akbar and Wymer, 2017); “credibility” and “reliability” (Fritz et al., 2017); and “consistency,” “continuity,” “integrity,” and “symbolism” (Eggers et al., 2013; Morhart et al., 2013). Brand authenticity is communicated through any modicum of origins, originality, uniqueness, or the unique manner in which the brand delivers on its promise (Schallehn et al., 2014). Creativity and personality are two sides of the same coin in brand authenticity. The study of Fritz et al. (2017) describes naturalness as an indication of “genuineness and realness.” A brand’s personality, originality, and naturalness are also described in a novel branding method and embedded in a genuine environment to differentiate from competitors.

With the development of the service industry, in marketing and publicity literature, the relevance of WOM management in consumer decision-making has been fully recognized. Earlier understanding of WOM was based on a dynamic communication process, a non-commercial communication behavior between the information receiver and the disseminator; that is, who tells what to whom (Herr et al., 1991; Stern, 1994). With the emergence of Internet media, which is said to open a new era of WOM (Hennig-Thurau and Walsh, 2003; Yeh and Choi, 2011), the definition of eWOM is “any positive or negative statement made by a potential, actual or previous consumer about a product or company, which is provided to many people and institutions through the Internet.” Compared with traditional WOM, eWOM has more comprehensive sources and more timely and efficient information (Sun et al., 2021), which is one of the reasons why this paper chooses to study eWOM intention.

The current literature focused mainly on the eWOM itself and its results, giving little consideration to the antecedent variables (Ahmad and Laroche, 2017; Ali et al., 2020; Golmohammadi et al., 2020; Sun et al., 2021). Therefore, this paper will fill in this gap by taking green brand authenticity as its antecedent variable (green attributes, quality, and integrity). As one of the possible factors the study of Fritz et al. (2017) discovered that brand authenticity influences the quality of brand relationships and consumer behavior intentions. When consumers perceive green brand authenticity, they will have positive behavioral intentions toward the green brand and also used to positive words while commenting on that specific brand. Such as positive reviews (Morhart et al., 2013; Moulard et al., 2016), the perception of brand authenticity will promote consumers’ positive support for eWOM intentions.

Therefore, we predict the following hypothesis:

*Hypothesis 1*: Green brand authenticity positively affects eWOM intention.

### Green Brand Authenticity and Brand Trust

Trust in several different studies refers to “the degree to which citizens have confidence in consistency, reliability, security, and integrity from an organizational, political, socio-economic, and technological perspective” (Mcknight et al., 2011; Shareef et al., 2015; Moulard et al., 2016; Shahzad et al., 2019, 2020; Ismagilova et al., 2020). A study by Munuera-Aleman et al. (2003) believes that brand intent has a more significant impact on consumers than brand reliability, because the latter may not constantly be a precise reflection of the true value of the brand. Consumers are more likely to trust brands that are honest and sincere than those that merely deliver quality. Building trust involves both high-quality service and a truthful approach (Schallehn et al., 2014). Some authors consider that consumers require a brand to meet their operational requirements, and trust is mainly developed by the emotional security that is increased by sympathy and reciprocity (Bakshi and Mishra, 2018). The study of Eggers et al. (2013) examined the associations between brand authenticity, brand trust, and small- and medium-sized enterprise growth and proved that overall brand authenticity fosters brand trust.

Furthermore, Eggers et al. (2013) also investigated that the respondents with high perceptions of authenticity reported significantly higher perceptions of brand authenticity than those with lower perceptions of authenticity. Trust is deemed to be a precondition for online transactions because e-commerce usually has a high degree of uncertainty, especially in social commerce (Nadeem et al., 2020). Most of the time, this may hinder the authenticity of the products and services of a particular brand. Therefore, trust is considered a prerequisite for brands to establish a long-term consumer relationship. Moreover, research on green brand authenticity and brand trust is empirically not tested by the previous researchers. In this view, we can assume that the authenticity of green brands may also have a positive effect on the development of consumer trust in a brand in an online buying context. Therefore, we posit the following hypothesis:

*Hypothesis 2*: Green brand authenticity positively affects brand trust.

### Brand Trust and eWOM Intention

Scholars have provided multiple definitions for trust, but there has been little consensus on trust (Ma and Orgun, 2006). Similarly, trust in e-commerce also turns out to have multiple manifestations (Gefen et al., 2003). Based on multidisciplinary knowledge, Munuera-Aleman et al. (2003) defined brand trust as “confident expectation of brand reliability and intention in case of risks to consumers.” Early research provided some insights; for example, Shirai (2017) revealed that price is the most decisive aspect because consumers with high price awareness are more likely to browse multiple Web sites to get the best price. In addition, Tsao and Hsieh (2012) found that if the Web site is not well designed, potential consumers will cancel the transaction when they visit a Web site to make a purchase. They affirmed that the Web site is the only platform where the company can convince potential visitors. Therefore, the Web site must have a professional appearance that reflects the firm’s overall competitiveness and products. E-commerce companies can increase consumer confidence by strengthening consumer trust toward their brand, thereby affecting consumers’ willingness to buy online (Rahman, 2020). It is possible to boost consumer confidence in eWOM if consumers have faith in online information and believe that it is reliable or legitimate, as has been demonstrated. A positive eWOM can enhance the positive attitude of the consumer toward a prescribed brand (Ahmad and Laroche, 2017; Rahman, 2020). Therefore, it is equally important in context of current study to measure the impact of brand trust in developing a positive eWOM intentions. Based on these studies, the following hypothesis was proposed:

*Hypothesis 3*: Brand trust is positively affecting eWOM intention.

Moreover, several studies accept that brand trust is a crucial determinant of consumers’ attitudes toward business relationships (Chaudhuri and Holbrook, 2001; Lude and Prügl, 2018), and marketers must become aware of its role in driving marketing success. In brand management, brand trust surpasses consumers’ satisfaction with product performance (Aaker, 1996). Therefore, developing and maintaining consumers’ online brand trust is crucial, especially for the development of e-commerce (Yeh and Choi, 2011). Brand trust is one of the key elements in a consumer-based brand performance model proposed by Molinillo et al. (2018), and it plays an indispensable role between enterprises and consumers. The studies proved that brand authenticity (Lude and Prügl, 2018) and perceived product quality (Han et al., 2019) could positively promote brand trust. Meanwhile, brand trust can further promote consumers’ brand loyalty (Morgan and Hunt, 1994; Durkan, 2020) and recommendation intention (Yeung et al., 2004). In this study context, the authors assume that brand trust can mediate the relationship between green brand authenticity and eWOM intentions. Therefore, the following hypothesis is offered:

*Hypothesis 4*: Brand trust plays a mediating role between green brand authenticity and eWOM intention.

### Moderating Effect of Self-Concept Consistency

Self-concept refers to the individual’s cognition and evaluation of self-attributes through self-experience (Sirgy et al., 1997). Self-concept consistency refers to the extent to which an individual’s self-attributes are consistent with his or her perception of things (in this case, green brand), and the degree of consistency varies from person to person. In the social media network environment, self-concept consistency plays an important predictive role (De Vries and Carlson, 2014). Consumers will self-certify through an authentic brand. When consumers’ self-image matches with brand image, self-concept consistency is considered a motivating factor for brand participation, leading consumers to participate more actively in the interaction with the brand (Morhart et al., 2013; Loureiro et al., 2017). A high level of consistency will increase enthusiasm for the brand in offline and online environments, such as recommendation intention (Kressmann et al., 2006; Usakli and Baloglu, 2011). The degree of consistency between self-image and green brand image will form different self-concept consistency among different people (Boucher, 2021). Different self-concept consistency will act on consumers, which will further affect consumers’ interaction with the green brand.

Trust is a kind of subjective consciousness influenced by self-cognition. As self-concept consistency increases, consumers’ subjective trust will increase accordingly. The closer the self-image and the brand image are, the more positive the consumer’s attitude toward the brand (Graeff, 1996; Boucher, 2011). If an organization commitment to stakeholders matches with its corporate principles, strategy, and articulated vision (examples include Apple, Porsche, etc.), it is said to be brand consistent. The distinctiveness of a firm is generated from its corporate values, which have grown over time as a result of the organization’s genesis (Eggers et al., 2013; Phung et al., 2019). Similarly, when consumers have a low green brand authenticity, those with high self-concept consistency may have more trust in the brand and are more willing to conduct positive eWOM than those with low self-concept consistency. Therefore, consumers with low self-concept consistency need more brand trust to consolidate their interaction with the green brand. In conclusion, we put forward that:

*Hypothesis 5*: Self-concept consistency negatively moderates the effect of green brand authenticity on brand trust.

*Hypothesis 6*: Self-concept consistency plays a moderating role in the mediating effect of brand trust.

**Figure 1:**
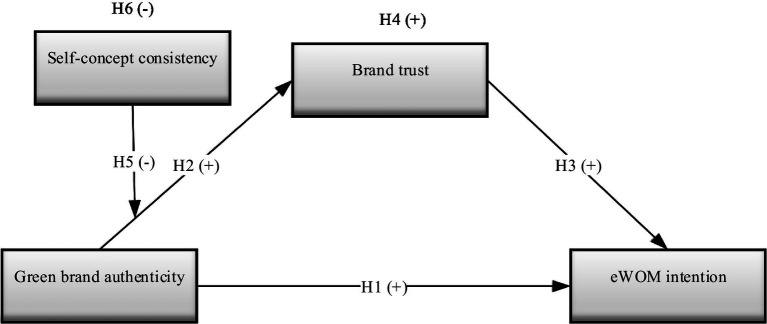
Research model.

To fulfill the aims of the research, a conceptual framework (see Figure 1) was developed.

## Research Methodology

### Sampling and Data Collection

This paper used an online questionnaire^1^ to assure respondents’ anonymity and confidentiality (Larson, 2019), which was filled out by consumers who had participated in online consumption. The authors use of online questionnaires to collect data, which can protect participants’ privacy to a certain degree, is considered the preferred way to avoid some adverse social reactions (Gittelman et al., 2015). In addition, without geographical location and time limitation, online questionnaires are featured by high efficiency and cost-saving (Lefever et al., 2007). Since the respondents were consumers who had participated in online consumption, it was more reasonable to use online questionnaires. Before answering the questionnaire, we gave the participants a questionnaire that provided the basic information and illustrations of a green brand that we designated in advance without knowing the brand name. The questionnaire included questions on respondents’ demographic information and a formal questionnaire that included the green brand authenticity scale, brand trust scale, eWOM intention scale, and self-concept consistency scale. Besides, before filling in this questionnaire, a question, “have you ever spread eWOM, such as purchasing reviews,” was designed to delete the respondents. If the response was no, the questionnaire was completed; if the answer was yes, the questioning was continued. Participants were provided essential information and illustrations of a product known to be a green brand with the brand name obscured in this stage of the formal questionnaire, and they were asked to complete the following scales based on the information.

Prior to the formal investigation, a preliminary investigation was carried out. A total of 96 questionnaires were collected in the pre-survey, and 91 valid questionnaires were finally obtained after eliminating the invalid questionnaires. According to the pre-survey analysis, the questionnaire was slightly modified to get the final formal questionnaire and circulated among the respondents during the month of January 2020. As of April 2020, among a total of 335 responses received, 292 cases have been analyzed due to 43 cases with default bias eliminated.

### Measures

To assure the reliability and validity of the scales used in this work, the measurements of the variables were based on maturity scales in relevant researches, and some items of the scales were modified after discussions with a team of experts. Participants’ response was measured using Likert’s 7-point method, from 1 (strongly disagree) to 7 (strongly agree). The following is the scale for each variable, and Table 1 shows the detailed elements for each measurement.

**Table 1:** Measurement model results.

Variable	Items		Factor loading	AVE	CR
GBA	Green Attribute	The brand is healthy, energy-efficient, and consumer-friendly	0.837	0.631	0.939
		The brand has made a certain amount of public welfare investment	0.806		
		The brand implements the concept of caring for the environment	0.743		
	Quality	The brand focuses on quality	0.829		
		The company strives to maintain long-term quality standards	0.744		
		The brand is well-made	0.816		
	Integrity	The brand will not violate its principles	0.780		
		The brand is faithful to the values it believes in	0.806		
		The brand has always been honest	0.784		
BT	I believe this brand		0.696	0.500	0.749
	This is an honest brand		0.806		
	This brand is safe		0.756		
WOM	If my friend wants to buy this product, I would like to share it with others using the “Share” function of the link		0.696	0.576	0.802
	If my friend is interested, I will introduce the content described by the brand when chatting online with him/her		0.801		
	If I find a forum with a topic similar to this product, I might write a review to recommend the product		0.774		
SC	The personality of the brand is consistent with how I see myself		0.697	0.730	0.931
	The personality of the brand reflects my image		0.835		
	People like me will use the brand		0.840		
	The brand reflects who I am		0.859		
	People who use the brand are very similar to me		0.857		

#### Green Brand Authenticity

The green brand authenticity scale was referred to the original brand authenticity scale (Napoli et al., 2016). Three dimensions with nine items were used in this scale: (1) green attributes with three items (e.g., “the brand is healthy, energy-saving, and consumer-friendly”), (2) quality with three items (e.g., “the brand is based on quality”), and (3) integrity with three items (e.g., “the brand remains true to its espoused values”). In this paper, the scale with nine items demonstrated good reliability (Cronbach’s *α*=0.905).

#### Brand Trust

Brand trust referred to the scale of Chaudhuri and Holbrook (2001), with a total of three items (e.g., “I believe in this brand”) measured with Likert’s 7-point. In this paper, the scale showed good reliability (Cronbach’s *α*=0.870).

#### eWOM Intention

Electronic word-of-mouth intention originates from the scale in Brown et al. (2005), which contained three items (e.g., “If my friends want to buy this product, I am willing to share it with them by using the sharing function of the link”). In this paper, the measure showed good reliability (Cronbach’s *α*=0.859).

#### Self-Concept Consistency

The self-concept consistency scale referred to the scale used by Malär et al. (2011), with a total of five items (e.g., “The brand’s personality reflects my image”). The measure showed excellent reliability (Cronbach’s *α*=0.919).

### Data Analysis Technique

A total of 292 valid answers were received, which met the requirement of 10 answers per item. In order to test the hypothesis, current research prefers PROCESS macro in SPSS rather than other covariance-based structural equation modeling (SEM) programs (Nivedhitha and Sheik Manzoor, 2020). Unlike the SEM program, it is simple and effective to perform mediation and moderation analysis, avoiding sample size limitations and degree of freedom. Moreover, it provides accurate statistical inferences, including specific conditional indirect effects and moderation–mediation indices, and avoids the problems caused by the interaction estimation provided by the latent variable method (Hayes, 2017). This paper used the PROCESS macro (Model 7) to test whether self-concept consistency moderates the mediation process. In addition, to check the indirect effects, we used the bootstrapping method (Hayes and Scharkow, 2013) to produce 95% bias-corrected confidence in-intervals from 5,000 resamples of the data effects would be significant when the confidence intervals excluded zero. Since this paper aimed to explore whether brand trust mediated the connection between green brand authenticity and eWOM intention and whether this mediation effect would be moderated by self-concept consistency, the analysis included the following three steps.

### Demographic Characteristics

Among the 292 participants, males accounted for 48.6 percent and females for 51.4 percent, indicating an even gender distribution. Results from Table 2, 45.5 percent of respondents are under the age of 25 and 35 percent are between the ages of 24 and 34years. Cumulatively, 80.5 percent of respondents are under the age of 35, suggesting that they may have a stronger proclivity to engage in online activities. We have asked the question about the Internet usage experience of the respondents. According to the findings, only 5.1 percent of respondents have used the Internet for shopping in the last 3years. Sixty-seven percent of those who answered the survey have been using the Internet for more than 5years. This will demonstrate to our audience that our participants are mature and well-versed in the usage of Internet. In addition, the authors gathered information on their educational background. The findings reveal that 51.9 percent of respondents have an undergraduate degree in their field. The fact that 31.3 percent of respondents had a graduate degree or higher demonstrates that participants are highly educated.

**Table 2:** Demographic information.

Characteristics	Statistic	
	*N*	Percentage (%)
***Gender***		
Male	142	48.6
Female	150	51.4
***Age***		
15–24years old	133	45.5
25–34years old	102	35
35–44years old	50	17.2
Over 44years old	7	2.3
***Internet usage experience***		
Less than 3years	15	5.1
3–5years	82	28.2
5–10years	131	44.7
More than 10years	64	22
***Education***		
Junior high school and below	14	4.8
High school/Technical secondary school	14	4.8
Higher vocational/Junior college	21	7.2
Proportion of undergraduates	152	51.9
Graduate or above	91	31.3

### Common Method Bias

To rule out the possibility of common method bias, the Harman single factor test was applied. The results indicated that the variance interpretation rate for the first component was 43, which is less than the acceptable value of 50 (Gentry and Calantone, 2002; Podsakoff et al., 2003; Kock, 2015). The findings indicated that there was no major common method bias in this study. Meanwhile, variance inflation factor (VIF) values were utilized to determine whether variables were collinear. The VIF values for each dependent and independent variable were determined to be 1.891, which is also less than the recommended value of 3.3 (Kock, 2015). On the basis of these findings, we can conclude that there was no significant issue of multicollinearity and common method bias.

## Results and Discussions

### Reliability and Validity Analysis

In this paper, the internal consistency coefficient Cronbach’s alpha was used for the reliability test. If Cronbach’s *α* value was above 0.7, the scale would have high reliability (Sekaran, 2006). By using SPSS 25.0 to test the reliability of the obtained data, it was found that the overall Cronbach’s *α* value of the questionnaire was 0.930, and the *α* value of each variable was above 0.85. Thereby, the internal consistency and stability of the scale were perfect, which met the recommended standards. In addition, six dimensions were extracted by principal component analysis of the scale, and the rotated component matrix was obtained. The factor loading of each item in the scale was according to the recommended values, with associated t-values for all indicators significant at *p*<0.001, indicating that the scale was magnificent (Table 1).

Validity includes content validity and constructs validity. The scales adopted in this paper all referred to the mature scales of existing research and adjusted based on consulting experts in related fields to ensure the content validity of the scales. Construction validity can be tested by convergence validity and discriminant validity. Generally, the average extracted variance (AVE) and composite reliability (CR) are used to measure the convergent validity of the scale. If the AVE value is above 0.5 and the CR value is above 0.7, the convergent validity of the scale is good (Hair et al., 1998, 2010). Using SPSS 25.0 to analyze the validity of the data, the results showed that the AVE value of the scale was in the range of 0.500 to 0.730, and the CR value of the scale was in the range of 0.749 to 0.939, indicating good convergence validity (Fornell and Larcker, 1981; Henseler et al., 2014). Discriminant validity was verified by Table 3, where the square root of AVE on the diagonal was greater than other values in its column. In addition, the overall KMO value of the questionnaire was 0.912, and the KMO of each variable was greater than 0.7. Bartlett’s test results rejected the null hypothesis at the 0.001 significance level. The results showed that the construct validity of the scale was very good.

**Table 3:** Descriptive statistics and bivariate correlations.

Variable	Mean	SD	1	2	3	4
GBA	4.706	1.070	**0.794**			
BT	5.112	1.209	0.687^*^	**0.707**		
WOM	4.779	1.363	0.668^*^	0.640^*^	**0.759**	
SC	4.493	1.101	0.290^*^	0.329^*^	0.354^*^	**0.854**

*N=292. SD, standard deviation*.

* *p<0.01. Diagonal values show the square root of AVE*.

*GBA, green brand authenticity; SC, self-concept consistency; BT, brand trust; and eWOM, electronic word-of-mouth intention. Inclined lines rendered in Boldface show the Square Root of the AVE of each variable*.

### Preliminary Analyses

The mean, standard deviation, and zero-order correlation coefficient of all variables were listed (see Table 3). The results found that consumers with higher green brand authenticity were more likely to have higher eWOM intention, and consumers with higher brand trust were also the same. Moreover, consumers who had a higher green brand authenticity were more likely to have higher brand trust. In addition, self-concept consistency and other variables showed a positive correlation. The hypothesis of this paper was preliminarily verified.

### Testing for Direct and Mediation Effect

Model 1, Model 4, and Model 5 were constructed to test the direct effect between green brand authenticity, brand trust, and eWOM intention (see Table 4). According to M1, the regression coefficient of green brand authenticity was 0.687 (*p*<0.001); that is, green brand authenticity significantly positively affected brand trust, which supported hypothesis 1. Similarly, green brand authenticity (*β*=0.668, *p*<0.001, M4) and brand trust (*β*=0.640, *p*<0.001, M5) had a significant positive impact on consumers’ eWOM intention, that is, hypotheses 2 and 3 were supported.

**Table 4:** Model testing.

Factor	BT			eWOM		
	M1	M2	M3	M4	M5	M6
GBA	0.687^**^	0.645^**^	0.630^**^	0.668^**^		0.433^**^
SC		0.143^**^	0.128^**^			
GBA*SC			−0.088^**^			
BT					0.640^**^	0.342^**^
*R* ^2^	0.471	0.490	0.497	0.447	0.409	0.509
Adj-*R*^2^	0.469	0.486	0.492	0.445	0.407	0.505
F	258.501^**^	138.773^**^	94.866^**^	234.197^**^	201.001^**^	149.621^**^

** *p<0.001*.

After examining the direct effect between green brand authenticity, brand trust, and eWOM intention, Model 6 was constructed to test the mediating effect of brand trust. According to M6, when the variable of brand trust was integrated based on Model 4, the *β* value of green brand authenticity on eWOM intention dropped from 0.668 to 0.433 (*p*<0.001). Meanwhile, brand trust significantly influenced eWOM intention (*β*=0.342, *p*<0.001, M6), indicating that hypothesis 4 was supported.

### Testing for Moderation–Mediation

**Figure 2:**
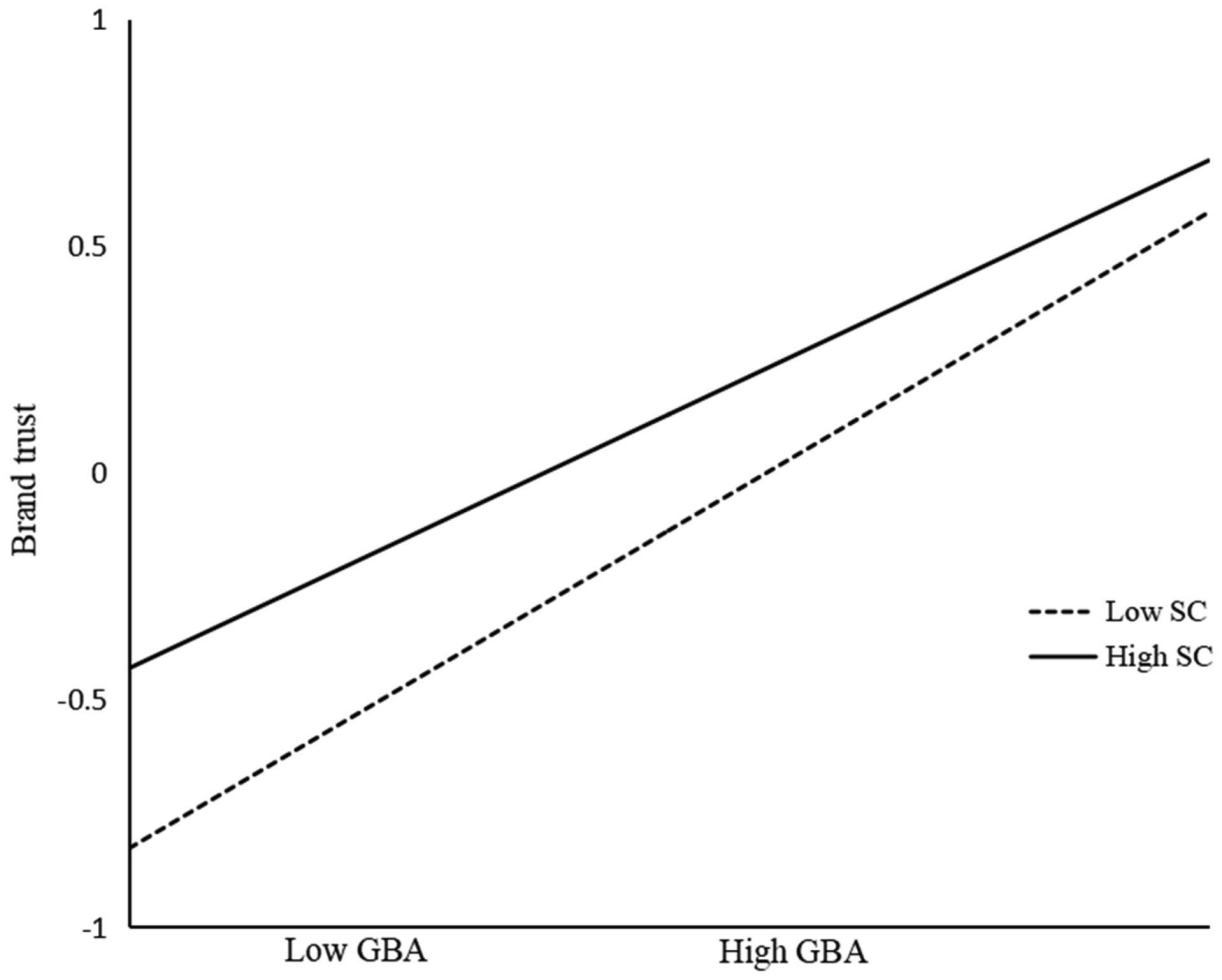
Moderating effects of self-concept consistency.

To test the moderating effect, Model 2 was built based on Model 1 after centralizing related variables. Meanwhile, Model 3 was further constructed by introducing the interaction effect. By analyzing the three models, self-concept consistency negatively affected the relationship of green brand authenticity and brand trust (*β*=−0.088, *p*<0.001, M3). From the diagram of the moderating effect of self-concept consistency (Figure 2), the slope of the association between green brand authenticity and brand trust was relatively higher for participants with lower self-concept consistency, whereas the slope was relatively weaker when the self-concept consistency of participants was high. With the increase of green brand authenticity, the increase of brand trust is even more significant, which supported hypothesis 5.

In order to test the moderation–mediation effect, the moderating effect of self-concept consistency and the mediating effect of brand trust were integrated using PROCESS macro (Model 7, bootstrap=5,000), and results are shown in Table 5. According to the results from Table 5, the confidence interval of self-concept consistency of different degrees did not contain 0 at 95% level, and green brand authenticity had a significant effect on eWOM intention through brand trust, which supported hypothesis 4 again. When self-concept consistency was low, the indirect effect of brand trust was 0.2397, but when self-concept consistency was high, the indirect effect of brand trust dropped to 0.1913. As self-concept consistency improved, the mediating role of brand trust in green brand authenticity and eWOM intention has gradually weakened; that is, the mediating effect of brand trust was negatively moderated by self-concept consistency. Hypothesis 6 was supported.

**Table 5:** Bootstrap analysis of significance test on moderation–mediation effects.

Moderator	Indirect effect	Standard error	95% confidence interval		Significance
			Bootstrap lower limit	Bootstrap upper limit	
Low self-concept consistency	0.2397	0.0527	0.1402	0.3463	Significant
Self-concept consistency	0.2155	0.0480	0.1273	0.3133	Significant
High self-concept consistency	0.1913	0.0474	0.1079	0.2919	Significant

### Major Findings

The study’s findings reveal that the authenticity of a green brand improves the brand’s eWOM intentions. This result is consistent with prior studies (Morhart et al., 2013), who suggest that when consumers perceive the authenticity of the green attributes, quality, and integrity of a green brand, they will put more confidence in the brand, thereby enhancing their willingness to be engaged in positive brand behavior. Especially, in the era when online social networks and sharing ideas are trendy, eWOM intention will be significantly improved. Additionally, as mentioned by Jumanazarov et al. (2020), consumer satisfaction can boost their intention to spread WOM; shaping the strength of a green brand appears to have a significant impact on consumer perception and behavioral decision-making.

Brand trust plays a mediating effect in the effect of green brand authenticity on eWOM intention as it directly reflects the quality of green brand authenticity. Studies have shown that higher brand authenticity will lead to higher brand trust (Lude and Prügl, 2018). According to Kim et al. (2009), once consumers have established brand trust, they are likely to have a good impression of the brand and have a positive intention to spread the WOM. When it comes to green brands, people are less likely to suggest them if they are not authentic. That said, it tends to be relatively clear that trust serves as the cornerstone of the good interaction between consumers and the brand, based on which brand manager can effectively predict their eWOM intention.

Furthermore, low self-concept consistency has a greater moderating effect on the overall mediating effect of brand trust. The theory of self-determination indicates that consumers with different self-concepts tend to show different degrees in accepting a brand. Since consumers with high self-concept consistency are more determined than consumers with low self-concept consistency, they are more likely to have trust in a high-fit brand. As a result, their impression of the brand’s objective attributes is reduced, while their intention to suggest the brand increases (Usakli and Baloglu, 2011), which is regarded as a positive WOM performance (Seo et al., 2020). As for consumers with low self-concept consistency who have relatively neutral attitudes toward the brand, authenticity can alleviate their uncertainty and help clarify their brand attitude, thereby generating brand trust in the brand. When green brand authenticity is low, high self-concept consistency serves as a protective factor to increase brand trust. However, as green brand authenticity gradually increases, low self-concept consistency has a more significant impact on the overall intermediary effect of brand trust.

## Conclusion, Implications, Limitations, and Future Prospects

### Conclusion

This empirical research examines the impact of green brand authenticity on eWOM intention from the consumer’s perspective. To that purpose, a quantitative methodology was used, and the conceptual model revealed that green brand authenticity was substantially connected with brand trust and self-concept consistency, indicating that it affected consumers’ eWOM intention. Additionally, brand trust was a critical mediator in determining consumer intention or eWOM and self-concept consistency was a critical moderator. Additionally, theoretical and managerial implications are discussed.

### Theoretical Implications

The structure of this paper and the key role of its function thus have been verified. That said, this paper provided a valuable theory, which was evidence-based. First, as for measuring brand authenticity, a good many brands researcher set different measurements from multiple dimensions (Schallehn et al., 2014; Akbar and Wymer, 2017; Fritz et al., 2017). This paper explains green brand authenticity through green attributes, quality, and integrity, thus enriching this analysis from the consumers’ perspective and providing a solid foundation for future endeavors. Second, brand trust plays a partially mediating role between green brand authenticity and eWOM intention, which could be considered as obvious evidence for the role of trust as a bridge between consumers and brands. The analysis of the mediating effect of brand trust in this paper expands the application of trust theory in green brand authenticity. Third, self-concept consistency has a negative moderating effect on the mediating effect of brand trust. Consumers with high self-concept consistency can be regarded as brand consumers, while low self-concept consistency can be regarded as non-consumers. The results of this paper reveal the difference between brand consumers and non-consumers, with the latter being more easily to be affected by green brand authenticity, which expands the theoretical development of consumer behavior.

In general, the theoretical contribution of this paper is to introduce the concept of green brand to the applicability of authenticity and to expand the research on the path of eWOM intention. Brand authenticity has an impact on the relationship between consumers and the brand (Fritz et al., 2017). This paper confirms for the first time that green brand authenticity contributes to the intention of eWOM. Second, it identifies and explains the mediating role of brand trust and the moderating effect of self-concept consistency in the cognitive-behavioral process, which contributes to the advancement of cognitive-behavioral theory in this area. In addition, the theoretical model of this article is adaptable for potential variables to be considered in the future. In this connection, the research provides a brand-new dimension for the research on green brand authenticity, which is very likely to lay a solid foundation for future sustainable development research.

### Management Implications

As the paper focuses on green brands in a relatively emerging market, our research results are helpful for a green brand manager find more effective promotion strategies. Measuring the authenticity of green brands reveals the importance of green attributes, quality, and integrity in ensuring green brand authenticity. Meanwhile, the green attribute trust, the quality of products will directly affect consumers’ perception and then consumers’ after-sales feedback. Integrity is an important guarantee for the long-term development of a brand. Brand dishonesty will directly affect brand authenticity and may even arouse consumers’ suspicion and distrust for the brand, especially for the green brand. In contrast, the quality of products will directly affect consumers’ perception and then consumers’ after-sales feedback. Therefore, finding the value of a green brand that consumers care about is conducive to shaping green brand authenticity.

The current goal of green brand construction is to maintain long-term, win-win relationships with consumers for greater consumer life cycle value. To do this, a green brand must gain consumer trust, which is critical to building consumer-brand connection (Eggers et al., 2013). So, authenticity is the perfect remedy for a brand trying to rebuild consumers’ trust. The green brand managers should attach greater importance to the mediating role of brand trust and actively enhance consumers’ trust in the brand. Through brand information disclosure, brand owners can maintain a relatively close relationship between consumers and green brands, which could also be strengthened through brand activities, both being effective ways to win back the consumers. In addition, brand after-sales service is also an effective way to build brand trust. Meanwhile, the Internet and social media are becoming more convenient in today’s transparent world, and small- and medium-sized businesses seeking to promote brand authenticity face significant obstacles. The current study model will assist policymakers in comprehending the fundamental relationship between the constructs and in developing policies appropriately to gain a competitive advantage. Based on the research findings, the green brand managers should not treat consumers with different self-concept consistency in the same way. For consumers with high self-concept consistency, a sound brand relationship should be maintained. Consumers with low self-concept consistency need to enhance brand authenticity, increase brand experience, and actively promote brand interaction. The results show that green brand authenticity has a positive impact on consumers’ eWOM intention. The manager should fully leverage the advantages of eWOM as an emerging force. That said, viral marketing must serve as an essential component of eWOM to gain greater exposure.

### Limitations and Future Prospects

This study offers some limitations and future research directions for researchers. First, the nature of this study was cross-sectional, and data were gathered from Chinese consumers using an online source of medium with small sample size. We suggest future research could take green brand authenticity and eWOM intention using longitudinal research design in Western samples to confirm the generalizability of the model. Second, while this work focuses on green brand authenticity and customer perceptions and confidence in various brands vary significantly, future research should shed additional light on various types of brands. Third, this study exclusively discusses the eWOM intentions, with no consideration given to offline WOM. It is also possible to consider the disparities in time and location between online and offline WOM and the differences in interpersonal intimacy between the two. Finally, this study has the potential to examine the usage of green branding for fraud, which may be covered in further research in the future.

## Data Availability Statement

The raw data supporting the conclusions of this article will be made available by the authors, without undue reservation.

## Author Contributions

YX: conceptualization, software, and writing—original draft preparation. YX and FS: methodology. JD and FS: validation. YX and XL: formal analysis and data curation. FS and XL: writing—review and editing. FS: visualization. JD: supervision, project administration, and funding acquisition. All authors have read and agreed to the published version of the manuscript.

## Conflict of Interest

The authors declare that the research was conducted in the absence of any commercial or financial relationships that could be construed as a potential conflict of interest.

## Publisher’s Note

All claims expressed in this article are solely those of the authors and do not necessarily represent those of their affiliated organizations, or those of the publisher, the editors and the reviewers. Any product that may be evaluated in this article, or claim that may be made by its manufacturer, is not guaranteed or endorsed by the publisher.

## Footnotes

^1^ www.wjx.cn
